# Exploring the Brazilian pediatric palliative care network: a quantitative analysis of a survey data

**DOI:** 10.1590/1984-0462/2023/41/2022020

**Published:** 2023-03-13

**Authors:** Esther Angélica Luiz Ferreira, Cristina Ortiz Sobrinho Valete, Silvia Maria de Macedo Barbosa, Graziela de Araujo Costa, Poliana Cristina Carmona Molinari, Simone Brasil de Oliveira Iglesias, Ana Cristina Pugliese de Castro

**Affiliations:** a Universidade Federal de São Carlos, São Carlos, SP, Brazil.; b Universidade de São Paulo, São Paulo, SP, Brazil.; c Hospital Sirio-Libanês, São Paulo, SP, Brazil.; d Hospital Beneficência Portuguesa, São Paulo, SP, Brazil.; e Faculdade de Medicina de Jundiaí, Jundiaí, SP, Brazil.; f Universidade Federal de São Paulo, Escola Paulista de Medicina, São Paulo, SP, Brazil.

**Keywords:** Palliative care, Medical education, Palliative medicine, Pediatrics, Delivery of health care, Community network, Cuidado paliativo, Educação médica, Medicina paliativa, Pediatria, Atenção à saúde, Redes comunitárias

## Abstract

**Objective:**

The aim of this study was to identify the characteristics of services in Brazil that compound the Brazilian Pediatric Palliative Care (PPC) Network.

**Methods:**

An online survey was conducted among representatives from PPC services. A total of 90 services from Brazil completed the online survey and answered a questionnaire about the service’s characterization, health professionals working in PPC, access to opioid prescription and education, and research in PPC.

**Results:**

In total, 80 services (88.9%) were created after 2010, 52 (57.9%) were in the southeast region, 56 (62.2%) were in public hospitals, 63 (70%) had up to 100 beds, and 57 (63.3%) were at the tertiary level. Notably, 88 (97.8%) had a physician in the team and 68 (75.5%) dedicated part-time to PPC. Also, 33 (36.7%) revealed concern with the care of health professionals and 36 (40%) reported difficulty or no access to opioid prescription. Research studies were reported to be conducted in 29 (32.2%) services.

**Conclusions:**

This mapping points out to a concentration of PPC services in the southeast region, with part-time professional dedication, and the need to improve professionals’ care. Difficulty in opioid access was reported. It is necessary to extend PPC participation to other Brazilian regions, increase time dedicated to PPC, improve professionals’ care and improve access to opioid prescription.

## RESUMO

**Objetivo:**

Identificar as características dos serviços que compõem a Rede Brasileira de Cuidados Paliativos Pediátricos (CPP).

**Métodos:**

Estudo do tipo *survey online* entre representantes de serviços de CPP. O total de 90 serviços do Brasil participaram da pesquisa e responderam a um questionário sobre a caracterização do serviço, os profissionais de saúde que nele atuam, o acesso à prescrição de opioides e a educação e pesquisa em CPP.

**Resultados:**

Oitenta serviços (88,9) foram criados após 2010, 52 (57,9%) estão na Região Sudeste, 56 (62,2%) em hospitais públicos, 63 (70%) têm até cem leitos e 57 (63,3%) estão no nível terciário. Oitenta e oito serviços (97,8%) tinham um médico na equipe e, em 68 (75,5%), os profissionais dedicavam parte de seu tempo aos CPP. Trinta e três serviços (36,7%) revelaram preocupação com o atendimento aos profissionais de saúde. Trinta e seis (40%) relataram dificuldade ou nenhum acesso à prescrição de opioides. Foi reportada a realização de pesquisas em 29 (32,2%).

**Conclusões:**

Este mapeamento aponta para a concentração dos serviços de CPP na Região Sudeste, com dedicação parcial dos profissionais, e para a necessidade de cuidar deles. Foi relatada dificuldade no acesso aos opioides. É necessário estender a participação na rede de CPP para outras regiões do Brasil, aumentar o tempo dedicado aos CPP pelos profissionais e cuidar destes, além de melhorar o acesso à prescrição de opioides.

## INTRODUCTION

The profile of pediatric patients has changed with the increasing need for assistance to children living with chronic and life-threatening diseases. In this scenario, Pediatric Palliative Care (PPC) emerged as an integral and respectful form of assistance to these patients and their families.^1^ PPC implies early identification, evaluation, and adequate treatment, improving quality of life, promoting dignity and comfort, without accelerating or delaying death, and may even positively influence the course of the disease, an essential aspect for the prognosis of the pediatric patient.^2,3^

According to the World Health Organization (WHO), in 2011, there were no PPC services in 65.6% of the countries surveyed.^1^ In 2019, in Brazil, 191 palliative care services were evaluated, and only 40.3% were qualified for the care of children and adolescents.^4^ In Brazil, some states have already published their own normative public policies about PPC.^5^ It is worth remembering that the specificities in pediatrics are unique and should be considered in the construction of an appropriate policy.^1,6^

Measuring the need and capacity to provide PPC are key elements in care planning of a country.^2^ In 2019, the International Children’s Palliative Care Network published estimated levels of provision of children’s palliative care worldwide, based on information available in the literature.^7^ These levels could range from 1 to 5, from lowest to highest, and Brazil was then classified as level 3, defined as “Evidence of localized palliative care provision for children and availability of training.”^7^

Networks are important to organize and measure demands. In the Netherlands, the creation of an integrated PPC network started in 2012 with the development of PPC teams and at first, and they analyzed what was needed.^8^ Also, PPC programs in Latin America have been considered underdeveloped when compared to other regions of the world, and the growth of a PPC network was suggested to organize the advocacy and help achieve the level of quality in PPC that children deserve.^9^

Less information is known about PPC in Brazil. Considering the latent need to map this, the aim of this study was to identify the characteristics of PPC services in Brazil, from the Brazilian Pediatric Palliative Care Network (BPPCN).

## METHOD

This descriptive, cross-sectional, online survey research is part of a larger study that investigates PPC in Brazil. Data were obtained through a questionnaire created by the researchers, who are the leaders of the BPPCN, based on their previous experience in a previous Brazilian overall palliative care mapping, and complemented information found in other studies and documents.^2,4,7,10,11^

The BPPCN was created in October 2020, as a collaborative research project developed by the Medicine Department of São Carlos Federal University, São Paulo. It is a formalized network that focuses on the promotion, spread, and dissemination of the knowledge in PPC and has a multidisciplinary nature. Institutions located in all Brazilian states participate and compound the study groups. The network leaders are professionals involved directly in assistance, teaching, and research in PPC in Brazil.

Online invitations were sent by BPPCN, in a snowball strategy, in different dates, through social media, to representatives of palliative care services operating in Brazil, which attended the pediatric age group. The online recruitment was made using a link to the questionnaire, in a Google Form, and occurred from February to May 2021. Only one representative per service was accepted. The sample was convenient. The inclusion criterion was being a palliative care service that attended to children. Exclusion criterion was a new entry from the same service or incomplete filling of the form.

The online survey questionnaire was composed of four structured components:

1. Identification and characterization of the services,
2. Characterization of health professionals,
3. Access to opioid prescription, and
4. Education and research, and a final section for commentaries, comprising 33 questions.

The questions were as follows: In which state was the service located?, When it was founded?, From which network the service was (private, public, philanthropic)?, Where did the service work (pediatric, general or oncologic hospital or homecare)?, level of complexity, binding to a university, if included a maternity, number of pediatric and neonatal beds, if they had beds exclusively for palliative care, how many beds were exclusively for palliative care, number of clinical offices for PPC, if there was a room for team meetings, if there was a pediatric intensive care unit and how many beds, if there was a neonatal intensive care unit and how many beds, if the PPC team was exclusively for palliative care, if the PPC team was exclusively for pediatrics, which professionals were included in the PPC team, what was the level of formation of the PPC team coordinator, what was the mean time dedicated to PPC during the week (<10 h, 10–30 h, >30 h), what was the level of PPC team care provision (inter-consultations, outpatient care, ward follow-up, telecare, home visits), who could make the first evaluation in an inter-consult, how many patients were attended in outpatient care per month, how many new cases were attended per month by the PPC team, which specialties forwarded more patients to PPC, if there was a pediatric residency program linked to the service, if there was grief support for families, which were the strategies for this support, level of access to opioid prescription (not accessible, accessible with difficulties, widely accessible), if there were teaching activities directed to PPC team, and how were these activities, if they developed research in PPC, if there were activities for the care of the PPC team, and how were these activities and final commentaries.

The questionnaire’s usability and functionality were previously tested among all researchers. To guarantee anonymity, the questionnaires did not demand the respondent’s personal name, but institution, and e-mail address. All IP addresses were checked for duplicates. Only the coordinator of the survey had access to all questionnaires’ answers. Data were stored in a database in a password-protected computer.

This study was approved by the Research Ethics Committee (CAAE 39915620.2.0000.5504) and all participants signed the Informed Consent Form.

Descriptive analyses were performed for the data of the online survey using Stata version 13.0 (Stata Corp, L.C.) and a self-developed data-extraction template. Results are presented as frequencies and 95% confidence intervals (95%CI), graphs, and tables.

The Checklist for Reporting of Survey Studies was used as the reporting guideline.^12^

## RESULTS

A total of 15 calls to participate were issued and 97 questionnaires were returned. All questionnaires were completely answered, and seven questionnaires were excluded because they were duplicated. The sample represented 90 PPC services, and all the questionnaires were completely responded. Regarding implementation, 10 services reported that they were created before 2009 and 80 (88.9%) after 2010.

Of these 90 services (Table 1), São Paulo was the state that had the majority of PPC services, with 38 services (42.3%), followed by Minas Gerais with 8 services (8.9%). Roraima, Pará, Goiás, Sergipe, and Rio Grande do Norte were the states with the lowest number, with one service registered in each of them (1.1% of the total in each state).

**Table 1.** Pediatric palliative care or palliative care services that serve the pediatric age group by states/Federal District of Brazil.

State	Number of services	Frequency (%)
São Paulo (SP)	38	42.3
Minas Gerais (MG)	8	8.9
Ceará (CE)	7	7.8
Paraná (PR)	6	6.7
Distrito Federal (DF)	5	5.6
Rio de Janeiro (RJ)	4	4.5
Santa Catarina (SC)	3	3.3
Pernambuco (PE)	3	3.3
Bahia (BA)	3	3.3
Maranhão (MA)	2	2.2
Mato Grosso do Sul (MS)	2	2.2
Espírito Santo (ES)	2	2.2
Rio Grande do Sul (RS)	2	2.2
Roraima (RR)	1	1.1
Pará (PA)	1	1.1
Goiás (GO)	1	1.1
Sergipe (SE)	1	1.1
Rio Grande do Norte (RN)	1	1.1
Total number of services	90	100

In this survey, the southeast represented the largest number of services, with 57.9% of the total (52 services), followed by the northeast, with 18.8% (17 services), the south with 12.2% (11 services), and the midwest with 8.9% (8 services). The north region had the lowest number of registered services, with 2.2% of the total (2 services).

The public network was represented by 56 services (62.2%; 95%CI 51.4–72.2) of services; 18.9% (95%CI 11.4–28.5) reported that they were exclusively in the private network and 8.9% (95%CI 3.9–16.8) were in the philanthropic network. Thirty-four (37.8%) services were in pediatric hospitals and 33 (36.7%) in general hospitals, 14 (15.5%) were home care services and only 5 (5.5%) were services in oncologic hospitals.

With respect to the number of beds, 37.8% (95%CI 27.8–48.6) reported that the hospital had up to 50 pediatric beds, 32.2% (95%CI 22.7–42.9) reported having between 50 and 100 beds, 10% (95%CI 4.7–18.1) between 100 and 200 beds, and 10% (95%CI 4.7–18.1) had more than 200 beds. Among these beds, it was also reported that only 13.3% (95%CI 7.1–22.1) were exclusive for PPC. Considering the presence of clinical offices for PPC, 55.6% (95%CI 50.4–72.1) reported that they were not available, 38.9% (95%CI 28.8%–49.7%) had up to 4 offices, and only 5.5% (95%CI 1.8–12.5) between 4 and 8 offices. The availability of rooms for team meetings occurred in 71% (95%CI 60.6–80.2) of the sites. Fifty services (55.5%; 95%CI 44.7–66.0) reported having a maternity unit, 67 (74.5%; 95%CI 64.2–83.0) a pediatric intensive care unit, and 55 (61.1%; 95%CI 50.2–71.2) a neonatal intensive care unit. Of the 90 services, 15 (16.7%) had homecare.

Three services (3.3%; 95%CI 0.7–9.4) reported being exclusively primary, 14 (15.5%; 95%CI 8.8%–24.7%) were secondary, 57 (63.3%; 95%CI 52.5–73.2) were tertiary, and 16 (17.8%; 95%CI 10.5–27.2) were quaternary level of attendance.

Most of the services provided care through inter-consultations (76.3%; 95%CI 65.3–84.0), 44.3% (95%CI 33.9–55.3) did it through outpatient care, 33.0% (95%CI 23.7–44.0) in ward follow-up, 25.9% (95%CI 16.9–35.8) by teleconsultation, and 20.6% (95%CI 12.3–29.7) by home visits.

Only 33 services (36.7%; 95%CI 26.7–47.5) reported having actions for the care of the team itself, including mental health groups, periodic mentoring meetings, meditation groups, and others.

Almost all the services had physicians (97.8%; 95%CI 92.2–99.7); most of them had psychologists (83.3%; 95%CI 74.0–90.3) and nurses (78.9%; 95%CI 69.0%–86.8%). Other results regarding the education of professionals are shown in Table 2.

**Table 2.** Multidisciplinary professionals working in Pediatric Palliative Care in Brazil.

Reported professional training	Number	Frequency (%)
Medicine	88	97.8
Psychology	75	83.3
Nursing	71	78.9
Social services	57	63.3
Physiotherapy	51	56.7
Nutrition	42	46.7
Occupational therapy	32	35.5
Speech therapy	29	32.2
Pharmacology	23	25.5
Odontology	14	15.5
Religious assistance	13	14.4
Physical education	1	1.1

Only 22 institutions (24.4%; 95%CI 16.0–34.6) reported having professionals that worked exclusively with the palliative care, while the remaining institutions (75.6%; 95%CI 65.3–84.0) showed their professionals splitting their time between PPC and the other specialties, 57 services (63.3%; 95%CI 52.5–73.2) providing PPC had professionals exclusively for pediatrics, and the remaining 33 services (36.6%; 95%CI 26.7–47.5) had professionals who cared for patients in various age groups.

The average time dedicated to the PPC was greater than 30 h per week in 5.6% (95%CI 1.8–12.5) of the services, 43.3% (95%CI 32.9–54.2) reported that this dedication was between 10 and 30 h per week, and 51.1% (95%CI 40.3–61.8) dedicated less than 10 h per week.

Considering health professionals’ complementary education, 42.3% (95%CI 31.9–53.0) reported that their coordinator had a *stricto sensu* postgraduation course, 56.7% (95%CI 45.8–67.1) had a *lato sensu* postgraduation course, 28.9% (95%CI 19.8–39.4) had medical or multiprofessional residence, and 27.8% (95%CI 18.8–38.2) were specialists in PPC.

**Figure 1.**
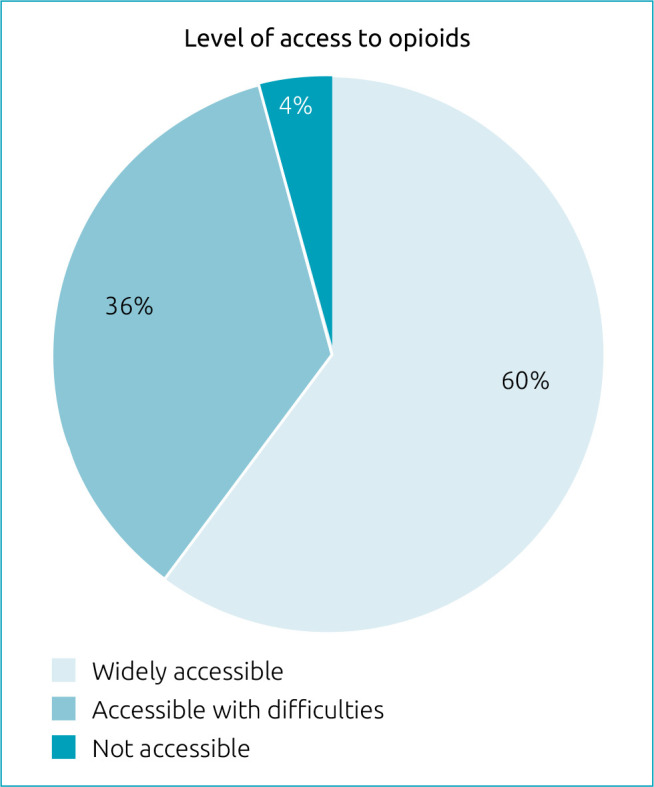
Level of access to opioids in the Pediatric Palliative Care services in Brazil.

Access to opioid prescription, when indicated, was reported as fully accessible in 60.0% (95%CI 49.1–70.2) of the services, and 40.0% (95%CI 29.8–50.8) reported having no access or difficulties, as shown in Figure 1.

**Figure 2.**
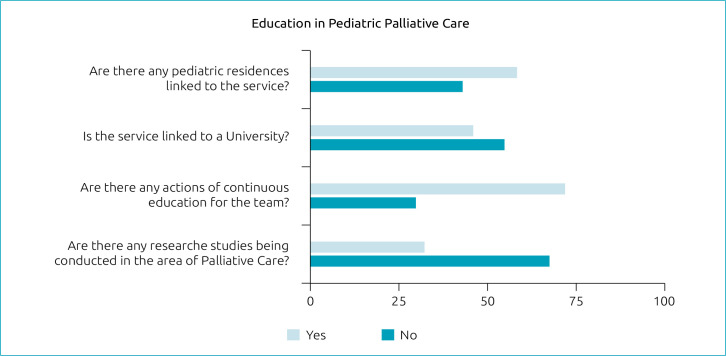
Health education in Pediatric Palliative Care services in Brazil.

In total, 51 services (56.7%; 95%CI 45.8%–67.0%) had pediatric residencies linked; one service reported having medical residency specifically in PPC, being one position per year. Few services were linked to some university. Of those, 15.6% (95%CI 8.8–24.7) were linked to a private university and 31.1% (95%CI 21.8–41.73) to a public university. Most (71.1%; 95%CI 60.6–80.2) of the services reported having continuing education actions in PPC. In 29 services (32.2%; 95%CI 22.7–42.9), research on PPC was developed (Figure 2).

## DISCUSSION

We identified an increase in PPC services in the last 10 years in Brazil. Most of the respondents were from the southern region. Some aspects seem to require improvement, such as the level of access to opioid prescription, the time dedicated to PPC, and attention to the care of health professionals.

Ninety services reported dealing with pediatrics. Although we cannot affirm how many service dealings with pediatrics exist in Brazil, we considered this an expressive sample. In 2019, 77 pediatric services had been pointed out in a survey conducted by the National Academy of Palliative Care, which suggests a steady growth.^4^ Most services were in São Paulo (42.2%) or in the southeast region (57.8%), which agrees with the results found in 2019, when 55.8% of all pediatric services were in the southeast region.^4^ We cannot affirm that other regions do not have PPC services, but we believe that the present survey reached institutions from all Brazilian regions. BPPCN is a young initiative, like other worldwide initiatives in the area.^8^

Most of the services (62.2%) were in the public health network. Brazilian data from a study on palliative care also revealed a predominance of public services, with 50% being exclusively public, 36% private initiative, and 14% mixed.^4^ We also found 8.9% of exclusively philanthropic institutions, but we did not find other studies to compare this result.

Services were linked more frequently to hospitals (77.8%), most of them (70%) with up to 100 beds, and on the tertiary level (63.3%). These results agreed with the literature. A study reported that adult and child palliative care services had a very variable attendance capacity, as only 12 services operated exclusively in hospitals and the number of dedicated beds varied up to 70 beds.^4^ These results suggested that there is a need to expand the PPC beyond the hospital scenario.

Almost all the services (97.8%) had physicians, 83.3% had psychologists, and 78.9% had nurses in the team. We emphasize that palliative care presupposes a multidisciplinary healthcare team. The different visions of the team in relation to PPC, as well as the complementary actions, make the integrality of care possible.^13^

It was reported that 60% of the services had access to opioid prescription, and 40% had some difficulty or no access. In 2019, considering all palliative care services in Brazil, only 7.9% reported having this difficulty, demonstrating that this may be even more critical when we refer to PPC.^4^ We must emphasize that symptom control is one of the PPC objectives. Also, pharmacological treatment is the pillar of pain control, and its adequate management is fundamental in palliative care, as this was reinforced by the WHO.^14^ Pain control is so important in palliative care in Latin America that some PPC services include pain in their name “Pain control and Palliative Care,” as reinforced by Zuniga-Villanueva et al.^9^ We understand that all PPC services should have free access to opioid prescription, when indicated.^15,16^ Therefore, we consider this 40% as an opportunity for improvement. Many factors could explain this result, such as the increase in opioids use and misuse in the past years, administrative subjects, and others. We advocate for opioid stewardship and, as suggested by Gaertner et al., although an approach for the judicious use of opioids must be done, in some countries, opioids are still insufficiently available even for patients with severe pain.^17^

Regarding the health team, most (75.5%) had partial dedication to PPC and 51.1% dedicated less than 10 weekly hours. In a Brazilian study on adult and child palliative care services, only 11% did not have exclusive physicians, as well as a considerable part of the services did not have nurses with exclusive dedication and only 33% had specific psychologists for the area.^4^ These results suggest that possibly in the children’s area, the difficulties in finding professionals exclusively dedicated to PPC may be even worse.

Regarding the well-being of the health team, it was observed that only 36.7% of the services reported this concern. We know that professionals who work with the process of death and dying are frequently exposed to heavy emotional and psychological burdens, which impact their quality of life.^11,18^ The literature suggests that the occurrence of burnout in these professionals is high and can reach up to 60%.^9,19^ For nursing professionals working with palliative care, it is estimated that the frequency of burnout is 24%, and it can be associated with occupational and psychological variables.^20^ Lack of personal accomplishment, emotional exhaustion, and depersonalization have been reported in affected professionals.^21^ Although we have not investigated burnout in the PPC team, we suggest that more attention should be given to health professionals’ well-being.

In this study, 32.2% of the services reported having research being carried out. Considering that most services were in hospitals with up to 100 beds, and that half of them had a pediatric residency program, it seems that most of them were focused primarily on care, instead of research. In the context of continuing education, identified by 71.1%, we suggest the need to intensify this issue, since it is essential that all professionals have the opportunity for in-service training, an orientation reinforced by the WHO.^2^ This statement becomes even more important when we analyze the scarce insertion of teaching in palliative care in the curricular guidelines of health courses, and the consequent deficiency in academic training at all levels of care, making continuing education an opportunity to rescue this demand.^22,23^ It has been suggested that health education for professionals working in PPC can provide the tools to minimize the effect of stress on their health and consequently on their work, including the use of coping strategies. In this way, besides qualifying the care offered by the professional, it helps professionals’ own health.^24^

This study has limitations. We did not calculate the response rate as we used a sensitive strategy to increase response, in social media: we had more responses than the number of services represented in the BPPCN. Also, as a preliminary mapping, we did not deeply investigate all the aspects involved in PPC. However, the results contribute to an evaluation of PPC in Brazil, cooperating to a better understanding of the theme in the national and international levels. The strengths of this study rely on its originality as the first report of BPPCN, the online characteristic of the survey, which reduces the interviewer bias, increases the response rate and validity regarding some sensitive subjects, and is quicker, as suggested by Sharma et al.^12^

Our study is the first to investigate the PPC services’ framework in Brazil. Brazil is a country with continental dimensions and disparities, and this was represented in our results. This highlights the need to spread PPC to other regions. National policies could be reinforced to guarantee the quality of care those children deserve.

Taking our results in a practical consideration, PPC respondent teams should investigate, at their local level, what is the difficulty in prescribing opioids. We could hypothesize that pain is still undertreated in some services, by the encountered difficulty – this will be the subject of our future survey. Although our study was limited to Brazil, other countries may also face the same difficulties.

The development of a national PPC network should be encouraged. We believe that this method is a powerful strategy to improve quality.

In conclusion, this mapping revealed that most of the PPC services in Brazil are recent, located in the southeast region, in public hospitals with up to 100 beds and of tertiary level. Almost all services have a physician on staff and most professionals dedicate part-time to the PPC, with half of them dedicating less than 10 h a week. Access to opioid prescription when indicated must be improved, just as the care for the health professional.
